# Apoptosis through Death Receptors in Temporal Lobe Epilepsy-Associated Hippocampal Sclerosis

**DOI:** 10.1155/2016/8290562

**Published:** 2016-02-23

**Authors:** Marcelo Ananias Teocchi, Lília D'Souza-Li

**Affiliations:** Centro de Investigação em Pediatria (CIPED), Faculdade de Ciências Médicas, Universidade Estadual de Campinas, Rua Tessália Vieira de Camargo 126, Cidade Universitária “Zeferino Vaz”, 13083-887 Campinas, SP, Brazil

## Abstract

Seizure models have demonstrated that neuroinflammation and neurodegeneration are preponderant characteristics of epilepsy. Considering the lack of clinical studies, our aim is to investigate the extrinsic pathway of apoptosis in pharmacoresistant temporal lobe epilepsy (TLE) associated with hippocampal sclerosis (HS) patients, TLE(HS). By a specific death receptor-mediated apoptosis array plate, 31 upregulated targets were revealed in the sclerotic hippocampus from TLE(HS) patients. Amongst them are the encoding genes for ligands (*FASLG*,* TNF, *and* TNFSF10*) and death receptors (*FAS*,* TNFRSF1A*,* TNFRSF10A, *and* TNFRSF10B*). In addition, we evaluated the hippocampal relative mRNA expression of the two TNF receptors,* TNFRSF1A *and* TNFRSF1B*, in patients, being both upregulated (*n* = 14; *P* < 0.01 and *P* < 0.04, resp.) when compared to the* post mortem *control group (*n* = 4). Our results have clearly suggested that three different death receptor apoptotic systems may be associated with the maintenance and progression of TLE-associated HS: (1) TNF-TNFRSF1A, (2) FASLG-FAS, and (3) TNFSF10-TNFRSF10A/B. Their effects on epilepsy are still scarcely comprehended. Our study points out to TNF and TNF receptor superfamily pathways as important targets for pharmacological studies regarding the benefits of an anti-inflammatory therapy in these patients.

## 1. Introduction

The connection between neurodegeneration and inflammation in the epileptic brain has emerged as an important axis for the comprehension of the pathomechanisms involved in seizure associated neuronal cell death. Hippocampal sclerosis (HS) is the main pathohistological abnormality found in resected tissue from temporal lobe epilepsy (TLE) patients. It is characterized by astrogliosis: an irregular proliferation of astrocytes due to the apoptosis of nearby neurons [1]. Several studies assert that inflammation has a crucial role in epileptogenesis [2], and there is a high probability that hippocampal chronic inflammation could exacerbate astrogliosis in TLE patients.

Apoptosis is divided into two main specific pathways: intrinsic and extrinsic. In spite of requesting particular triggers to start a cascade of molecular events, both pathways converge in the activation of CASP3, and both are present in TLE-associated HS. The apoptotic intrinsic signaling pathways are initiated by non-receptor-mediated intracellular signals (e.g., DNA damage, radiation) that result in changes in the inner mitochondrial membrane. Seizures by themselves can represent an interesting example of these kinds of signals. One of the primary events in hippocampal seizure-induced apoptosis is the excessive release of glutamate with consequent intracellular calcium overload, culminating in downstream swelling and rupture of intracellular organelles and activated proteolytic enzymes leading to cell death [3].

Unlike the intrinsic signaling pathways that initiate apoptosis, the extrinsic signaling pathways involve transmembrane receptor-mediated interactions. These include death receptors which are members of the TNF receptor gene superfamily [4]. Apoptotic or survival signals are the consequence of the death receptor family activation by death ligands. Various members of this family have been described thus far, including TNFRSF1A, FAS, TNFRSF25, TNFRSF10A, TNFRSF10B, and TNFRSF21 [5]. The execution pathway can be initiated by these receptors through CASP8 or CASP10 whose activation could trigger the proapoptotic proteins BID or BAX, via TP53, culminating in a cross talk with the mitochondrial or intrinsic pathway of apoptosis [4].

Recurrent seizures induce not only neuronal cell loss but also inflammation [6]. However, neuronal cell loss is not a prerequisite for inflammation to happen; rather, the liberation of proinflammatory cytokines can contribute to cell death [7], and dying cells may perpetuate inflammation [6]. Previously, we reported a marked TNF upregulation in TLE(HS) patients [8], which is indicative of chronic hippocampal inflammation.

We believe that the neuronal apoptosis through death receptor pathways highlights the key molecular events involved in triggering astrogliosis and TLE-associated HS. Understanding which are the soluble mediators and the molecular mechanisms crucially involved in the link between inflammation and neuronal cell death is instrumental to shed light on how seizures may contribute to HS in epilepsy and to identify new therapeutic targets for the treatment and cure of pharmacoresistant epilepsy, particularly in TLE(HS) patients.

## 2. Methods

### 2.1. Subjects and Tissue Collection

Ethical approval was certified by the “Comitê de Ética da Faculdade de Ciências Médicas da Unicamp” (CEP n 470/2003). Subjects' data was reported in a recent study [8]. The present study was performed with the same patient and* post mortem* control samples. Briefly, TLE and HS were detected by telemetry/video electroencephalogram and magnetic resonance image, respectively, in 14 patients. Due to the pharmacoresistance of the syndrome, they went through an amygdalohippocampectomy for treatment. All hippocampal tissue samples were immediately collected and divided into two parts. One portion was fixed for histopathological analysis and HS/astrogliosis was confirmed in all of them [H&E staining and the terminal deoxynucleotidyl transferase dUTP nick end labeling (TUNEL) assay; data not shown]. The second portion was immediately snap-frozen in liquid nitrogen after surgery and stored at −80°C until RNA isolation.

Four* post mortem* control hippocampal tissue samples (1 female, 3 males; 22.75 ± 5.56 years old, ranging from 19 to 31) were kindly provided by the “Instituto Médico Legal, IML” (Forensic Institute) of Campinas.* Post mortem* control subjects passed away instantaneously or quickly. Although their deaths were traumatic, which runs against the occurrence and progression of neuroinflammation, neurological findings were not detected and the* post mortem* delay averaged 7.8 h (range: 6.0–9.0 h).

### 2.2. Gene Expression

All reagents were purchased from Thermo Fisher Scientific Inc.; Waltham, MA 02451, USA (former Life Technologies; Foster City, CA 94404, USA).

#### 2.2.1. RNA Extraction, Array Gene Expression, and RT-qPCR

Total RNA extraction and RT-qPCR were carried out according to our previous work [8]. TRIzol^®^ Reagent was used for RNA extraction according to the manufacturer's instructions. The RNA integrity number (RIN) average in control and patient groups was 7.525 ± 0.5437 and 6.155 ± 0.2484, respectively. Afterwards, 1 *μ*g of total RNA of each sample was reverse transcribed into cDNA using 200 U of Superscript^®^ III Reverse Transcriptase and 3 *μ*g of Random Primers. Sterilized and filtered DEPC treated water was used in all RNA procedures.

To identify potential targets associated with apoptosis through death receptor signaling, we used a TaqMan^®^ Array Human Apoptosis through death receptors 96-well plate (PN: 4414105). The plate contained 44 assays of genes associated with death receptor-mediated apoptosis and four assays of reference gene (endogenous control) candidates (Table 1). Gene names are in accordance with the approved symbol from the HUGO Gene Nomenclature Committee (HGNC) database. All assays were plated in duplicates: one for cDNA pooled from the TLE(HS) patients (*n* = 12) versus the other for cDNA pooled from the* post mortem* controls (*n* = 4). Among several factors which can influence *C*_*T*_ (cycle threshold) and consequently qPCR results credibility, we emphasized instrument calibration and cDNA quality. The template concentration (e.g., concentration of total RNA converted to cDNA) should always be homogeneous among samples, particularly when working with pools. cDNA concentration and purity were assessed by spectrophotometry (NanoDrop ND1000; NanoDrop Technologies, Wilmington, DE). The cDNA concentration means were 858.1 ± 30.07 and 871.9 ± 27.10 for control and patient samples, respectively. cDNA absorbance (A260/A280) ranged from 1.84 to 1.90 considering all 16 samples. Reactions were carried out according to manufacturer's instructions. We used a total volume of 20 *μ*L, comprising 10 *μ*L of TaqMan Gene Expression Master Mix (Life Technologies) and 10 *μ*L of pooled cDNA diluted in DNase-free water. The final concentration of the pooled cDNA samples used was 10 ng in 10 *μ*L for each 20 *μ*L PCR reaction.

Two target genes were chosen to be tested separately by subject:* TNFRSF1A* and* TNFRSF1B* (Assay ID: Hs00153550_m1). The latter was not included in the array plate. The PCR efficiency validation was also performed according to our previous work [8]. The amplification efficiencies were close to 1.0 (100%). cDNA samples derived from the investigated genes were detected using an ABI PRISM^®^ 7500 Sequence Detection system and TaqMan Gene Expression Assays. For RT-qPCRs, reference genes were selected according to Wierschke et al. [9]. Therefore,* HPRT1* and the geometric mean of* ENO2* and* TBP* were used as our normalization factors. Both were indicated as being the most stable reference genes in epileptogenic tissue [9]. Each qPCR was run as triplicates with 10 ng cDNA sample in 6.25 *μ*L TaqMan Gene Expression Master Mix, 0.625 *μ*L of the respective probe/primer mix and 0.625 *μ*L purified and deionized H_2_O. All reactions were run as triplicates, and measurements with a difference of more than 0.3 *C*_*T*_-values were excluded from analysis.

### 2.3. Data Analysis

Relative gene expression data was generated and analyzed by the 7500 Software version 2.0.5 (Life Technologies). The software GraphPad Prism 5 was used for the statistical analysis (GraphPad Prism version 5.04 for Windows, GraphPad Software, San Diego California USA, *〈* http://www.graphpad.com *〉*). The Mann-Whitney *U* test was used for comparison between data from the control group (*n* = 4) versus the patient group (*n* = 14). For all analyses, differences of *P* < 0.05 were considered significant.

A molecular pathway related to differentially expressed genes was created by the IPA-Ingenuity Pathway Analysis (Ingenuity Systems, *〈* http://www.ingenuity.com *〉*) application via the “Core Analysis” function. The array data sheet generated by the 7500 Software was uploaded into the IPA application. The differentially expressed genes were connected based on the reported association among genes or proteins and their functional roles.

## 3. Results

**Figure 1:**
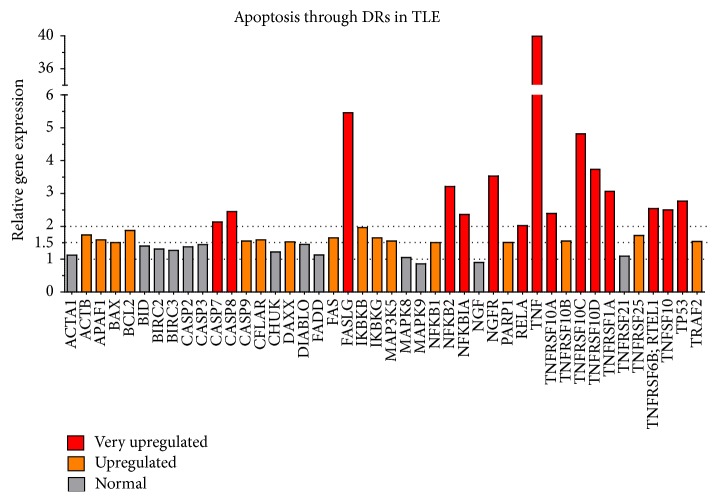
Hippocampal expression of genes associated with apoptosis through death receptors in TLE(HS) patients versus* post mortem* controls. The combination of* GAPDH* and* HPRT1* was used as the reference gene. The pool of* post mortem* control samples (*n* = 4) was the reference sample (calibrator) and its relative quantification (RQ) was always 1.0. Different colors represent gene expression in RQ values (or fold-change values, when RQ is a positive number) and correspond to the following parameters: red = very upregulated (RQ ≥ 2.0); orange = upregulated (1.99 ≥ RQ ≥ 1.5); gray = normal (1.49 ≥ RQ ≥ 0.67). None of the 44 target genes analyzed was downregulated (0.66 ≥ RQ ≥ 0.51) or very downregulated (RQ ≤ 0.5).

Array gene expression quantifications are shown in Table 1 and Figure 1. From 44 target genes associated with apoptosis through death receptors, we found 31 upregulated genes (70.45%). Our criterion to define an upregulated gene was based on an increase of a minimum of 50% on mRNA levels in comparison to controls, which corresponds to a cutoff of 1.5-fold change. Choosing a precise cutoff is always a challenge in array studies and it is crucial to analyze results and select the most important targets. Considering our previous study on* NFKB1* expression in the same group of TLE(HS) patients (*n* = 14) [8], a 1.5 cutoff is statistically acceptable since the* NFKB1* expression in the array was exactly 1.5-fold.

The number of upregulated genes is narrowed to 15 if a cutoff of 2.0 is considered (at least an augmentation of 100% on mRNA levels). This more conservative cutoff reveals critical target genes that can be divided into 3 categories: (1) ligands and receptors, (2) mediators, and (3) molecules related to the transcription factor nuclear factor kappa B (NFkB). Discussion will be guided by these categories and focused on the controversies and novelties related to these most upregulated targets.

**Figure 2:**
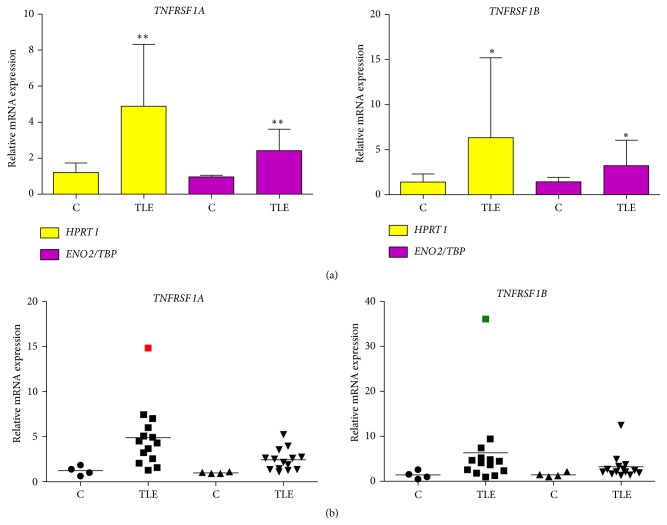
Hippocampal gene expression of* TNFRSF1A* and* TNFRSF1B* in TLE(HS) patients versus* post mortem* controls.* HPRT1* and* ENO2/TBP* were used as reference genes. One of the* post mortem* control samples was randomly chosen as the reference and its relative quantification was always 1.0. All quantification data for the remaining samples, including controls and patients, was benchmarked to the reference sample. Samples were separated in two groups: TLE(HS) patients (*n* = 14) and* post mortem* controls (*n* = 4). (a) Columns are means with SD. Mann-Whitney *U* tests were used for the comparison between groups. ^*∗*^ *P* < 0.05; ^*∗∗*^ *P* < 0.01. (b) Marks are different geometric figures, which represent the gene expression mean of the samples. The horizontal line is the mean of the group analyzed. Circles and squares correspond to gene expression with* HPRT1* as the reference. The two kinds of triangles correspond to gene expression with* ENO2/TBP* as the reference. The red and green squares (outliers) correspond to TLE 09 and TLE 03 patients, respectively (see Table  1 in Teocchi et al., 2013 [8]).

**Figure 3:**
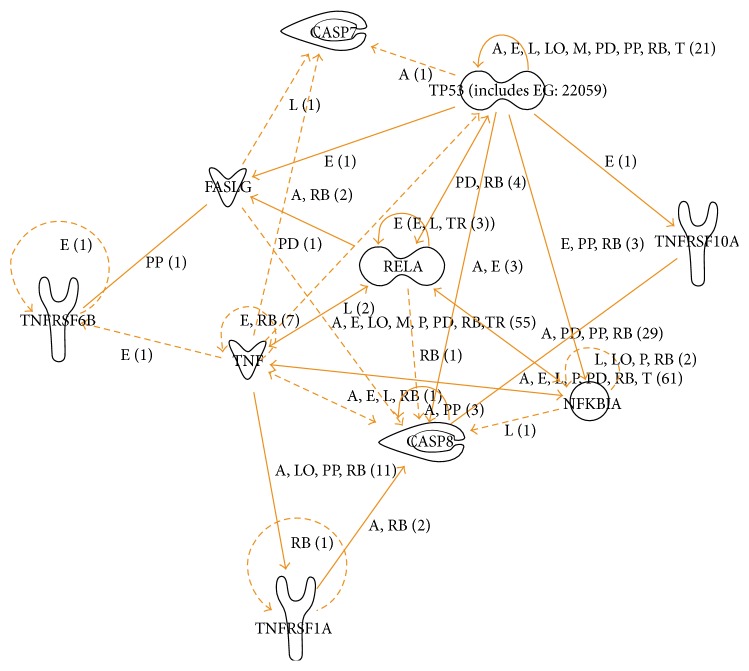
Apoptosis through death receptors: a signaling pathway in TLE(HS). This pathway was generated by the IPA software, based on 10 of the 15 upregulated genes (cutoff 2.0-fold change; see Table 1) in hippocampal tissues from TLE(HS) patients (*n* = 12) compared to* post mortem* controls (*n* = 4). The biological relationship between two genes, represented as nodes, is shown as a line. Nodes with different shapes indicate different functional class. The pathway created did not include the following genes:* NFKB2*,* NGFR*,* TNFSF10*,* TNFRSF10C,* and* TNFSF10D*. The function “nervous relaxed” was used. A, activation; E, expression regulation; I, inhibition; L, proteolysis; LO, localization; M, biochemical modification; P, phosphorylation; PD, protein-DNA interaction; PP, protein-protein interaction; RB, regulation of binding; T, transcription; and TR, translocation.

Regardless of the cutoff or even the reference genes, array* TNF* expression (39.96-fold) was very meaningful, particularly in comparison to the expression of the other genes. This significant overregulation led us to quantify separately the expression of its receptors,* TNFRSF1A* and* TNFRSF1B*, being both statistically upregulated (*P* < 0.01 and *P* < 0.04, resp.) (Figure 2). In Figure 3, we proposed a schematic pathway which involves TNF, TNFRSF1A, and eight of the other 13 very upregulated genes.

## 4. Discussion

Inflammation, degeneration of neurons and HS are closely related [10]. Recently, we reported a marked* TNF* upregulation in TLE(HS) patients [8], which is indicative of chronic hippocampal inflammation. The importance of this cytokine as one of the main apoptosis “propellers” in TLE-associated HS is reinforced by our Human Apoptosis through death receptors array results (Table 1 and Figure 1) which show the upregulation of 31 related genes. They are associated with the extrinsic apoptosis signaling pathway, among them members of the TNF and TNF receptor gene superfamilies and several factors which play a role in the cell death cascade. The vast majority of them have never been quantified in patient's samples. Below, we present and discuss our finding which suggests the involvement of these genes with neurodegeneration and the consequent astrogliosis in TLE(HS).

### 4.1. Apoptotic Systems: Ligands and Receptors

#### 4.1.1. The TNF-TNFRSF1A Axis

There are few clinical studies on the TNF system and its effects on epilepsy in spite of intense investigation in animals. Several models indicate that seizures induce the TNF expression in the brain [2, 11–14] although some have shown no important TNF alterations in plasma or CSF after different kinds of seizures [15–18]. In patients, Sinha et al. detected increased serum levels of TNF and other cytokines in several epilepsy syndromes [19]. Our results for TLE(HS) patients differ from those on seizure models only by the fact that the* TNF* mRNA increase does not seem to be transiently enhanced. Some patients had their last seizure several days before the surgery, implying that the high* TNF* expression levels were happening often, suggesting that chronic hippocampal inflammation could be intrinsic to refractory TLE(HS).

The most controversial evidence on the TNF role in epilepsy was raised when Balosso et al. reported that intrahippocampal injection of murine-recombinant TNF in mice potently prevented seizures [20]. Moreover, transgenic mice overexpressing TNF by astrocytes showed shorter seizures, whereas deficient mice for TNF receptors had prolonged seizures [20]. The dichotomic effect of TNF on seizures may be related to the tissue microenvironment, including the cellular source (neuronal versus glial) of TNF release, the extent of TNF increase, its concentration and persistence in tissue, and action through its two receptors expressed by the targeted cells [14, 20, 21].

In the hippocampus, TNF is able to activate its two receptors (TNFRSF1A and TNFRSF1B) to regulate cell-signaling pathways [22, 23]. TNFRSF1A is ubiquitously expressed in human tissues and is the principal signaling receptor for TNF. A cytoplasmic death domain, required for apoptotic signaling pathways and NFkB activation, is present in this major receptor. TNFRSF1B does not contain a death domain and actuates in restricted biological processes, being mainly expressed in immune cells. Through intracellular adaptors called TRAFs (TNF Receptor-Associated Factors) associated with this receptor, JNK (c-Jun N-terminal kinase) is activated during the cell survival induction through activation of NIK (NFkB-inducing kinase), a downstream target of TRAF2. Thus, TNFRSF1A has been involved in apoptosis activation, whereas TNFRSF1B is implicated with activation of the NFkB system [24–26]. Likewise, seizure model studies imply that the TNFRSF1A pathway is associated with deleterious effects and that the TNFRSF1B pathway is related to anticonvulsive outcomes [14, 27].

Our hippocampal RT-qPCR results showed the upregulation of both TNF receptors in TLE(HS) patients (Figure 2). The deleterious consequences of TNFRSF1A action are well-known; however, for TNFRSF1B, consequences are still unclear. TNFRSF1B could be triggered as a survival mechanism to compensate for the extensive neuronal cell death found in epilepsy-associated HS or its expression could enable the harmful consequences of TNFRSF1A activation [28]. These authors demonstrated that TNFRSF1B significantly decreased the TNF concentration required for cell death without the generation of an intracellular signal. Instead, TNFRSF1B modulated the rate of TNF association with TNFRSF1A, possibly by increasing the local concentration of TNF at the cell surface through rapid ligand association and dissociation. Functional experiments are necessary to verify this finding in brain cells and clarify the role of TNFRSF1B in TLE(HS). Furthermore, these authors proposed that other cell-surface receptors, such as NGFR, may utilize an analogous “ligand passing” mechanism. Our array results showed the upregulation of* NGFR* (3.53-fold). Increased expression of NGFR in hippocampal neurons of TLE patients has already been described [29].

#### 4.1.2. The FAS-FASLG Axis

FASLG triggers apoptosis by binding to FAS [30]. Our finding showed the upregulation of both* FAS* (1.65-fold) and* FASLG* (5.46-fold), which represents an important apoptotic system scarcely investigated in clinical cases. Only one study reported an augmentation of FAS in the sclerotic hippocampus from TLE patients [31]. In animals, seizures induced a significant augmentation in FAS expression within the ipsilateral hippocampus from 4 to 24 h after levels returned to baseline [32]. Similarly, increased expression of both* Fas* mRNA and protein were evident in the adult rat brain from 4 h to 5 days after the onset of kainic acid-induced seizures and neurons with increased FAS expression were also immunoreactive for TP53 [33]. Induced by kainate, FASLG expression increased rapidly at 6 h and returned to the basal level at 3 days in CA1 and CA3/DG (dentate gyrus) hippocampal regions [30, 34].

*FASLG* was the second most upregulated gene according to our results (Figure 1). Considering the chronic status of our patients, this cytokine does not appear to have a transient expression in pharmacoresistant TLE(HS) and might contribute to neuronal loss, being the FAS-FASLG pathway an interesting target for pharmacological studies. In addition, we found that* TNFRSF6B* was also overregulated in TLE(HS) patients (2.54-fold). The encoded protein by* TNFRSF6B* is assumed to play a regulatory role in suppressing FASLG-mediated cell death. It acts as a decoy receptor that competes with death receptors for ligand binding [35] (Figure 3). We have not found any studies regarding the association between TNFRSF6B expression and epilepsy.

#### 4.1.3. The TNFSF10-TNFRSF10A/B Axis

Besides apoptosis mediated by TNF and FASLG and its receptors, TNFSF10 is another potent inducer of apoptosis [36, 37]. Both apoptosis-inducing and nonapoptosis-inducing membrane-bound receptors have been described for TNFSF10. Only TNFRSF10A and TNFRSF10B are able to generate a death signal [38, 39], while TNFRSF10C and TNFRSF10D are truncated and have been proposed to function as decoy receptors by binding TNFSF10 without producing a death signal, thereby inhibiting apoptosis [39, 40]. Our results on gene expression accentuate the importance of TNFSF10 (2.50-fold) and its upregulated receptors (Figure 1). They have never been quantified by RT-qPCR in human TLE hippocampal samples. In brain tissues from TLE patients, Dörr and colleagues did not detect the expression of TNFSF10, but both apoptosis-inducing and non-apoptosis-inducing receptors were expressed on neurons, astrocytes, and oligodendrocytes, which indicate their possible susceptibility to TNFSF10-mediated apoptosis [41]. On the other hand, a significant increase on TNFSF10 expression in both patients and animals (seizure model) has been reported [42]. In accord with our results, it is conceivable that there is an important action of TNFSF10 and its receptors in TLE(HS)-associated neuronal apoptosis. The expression and regulation of these receptors might be crucial for death or survival of an individual cell, since both apoptosis-mediating and apoptosis-blocking receptors were present on the different brain parenchymal cells [37, 41]. Our data per se shows an overview on this apoptotic system and is complemented by the two previously discussed studies on proteins.

### 4.2. Apoptotic Mediators: Caspases and TP53

We found elevated mRNA levels for* CASP7*,* CASP8,* and* CASP9* in our TLE(HS) samples (Table 1 and Figure 1). This finding extends and reinforces protein data from seizure models and human epileptogenic tissue studies, which have reported a deregulated expression of caspases [32, 43–46]. It is well-known that sequential activation of caspases plays a central role in the execution phase of cell apoptosis.

CASP7 is an effector, responsible for cleaving important apoptotic intracellular substrates. Yamamoto et al. found X-linked inhibitor of apoptosis (XIAP) binding CASP7 in TLE brain, suggesting ongoing antiapoptotic responses, which might be impeding caspases from inducing apoptosis [46]. XIAP can only bind activated CASP7 to modulate its activity [47], and XIAP expression is regulated after experimental seizures [48]. Their finding reinforces the evidence for active CASP7 in the TLE hippocampus. CASP8 is a key initiator of apoptosis via death receptor-mediated pathways, capable of inducing apoptosis by directly processing executioner caspases [32]. In a seizure model, its inhibition significantly reduced neuronal apoptosis, accompanied by a decrease of tBID (truncated BID), cleaved CASP9, and cytosol cytochrome c [45]. Our results are in agreement with the studies mentioned and emphasize the importance of CASP7 and CASP8 in TLE(HS) (Figure 3).

A* Tp53* overexpression occurred along with excitotoxicity in an adult rat brain within hours after systemic administration of glutamate analogue kainic acid [49]. Years later and after several other reports on altered TP53 expression in seizure models, including the already mentioned study of Tan and colleagues [33], Xu et al. found TP53 positive cells in the sclerotic hippocampus from TLE patients [31]. In the same year, Engel and colleagues detected significantly higher levels of TP53 by Western blotting in hippocampal tissues from TLE patients [50]. Our result on the elevated* TP53* mRNA level extends and reinforces this data. It is still unclear if TP53 could function as a potential target for protection in seizure-induced neuronal death since TP53 regulates a large number of genes, which would need to be carefully evaluated [31].

### 4.3. Subunits and Regulators of the Transcription Factor NFkB

As previously reported, we found that* NFKB1* was upregulated in TLE(HS) patients [8]. In the present study, our array results showed that* NFKB1* was 1.5-fold more expressed than controls. Several other genes related to the NFkB complex, such as* IKBKB* (1.96-fold),* IKBKG* (1.65-fold),* NFKB2* (3.21-fold),* NFKBIA* (2.36-fold), and* RELA* (2.02-fold) also showed an increased expression (Figure 1). Taken together, our finding clearly emphasizes the NFkB involvement in TLE(HS) physiopathology (Figure 3). In addition, protein or mRNA expression data on those molecules is scarce, even from seizure models.

A number of signal transduction events, initiated by inflammation, immunity, differentiation, cell growth, tumorigenesis, and apoptosis, converge on NFkB activation [51]. This pleiotropic transcription factor, present in practically all cell types, is a homo- or heterodimeric complex formed by the Rel-like domain-containing proteins RELA/p65, RELB, NFKB1/p105, NFKB1/p50, REL, and NFKB2/p52. The heterodimeric p65-p50 complex appears to be the most common. NFKBIA and NFKBIB (I-kappa-B proteins) inhibit the NFkB complex by trapping it in the cytoplasm [51]. The kinases IKBKA and IKBKB mark I-kappa-B proteins for destruction via the ubiquitination pathway, thereby allowing activation of the NFkB complex. IKBKG is a regulatory subunit of the inhibitor of kappa B kinase (IKK) core complex which phosphorylates NFkB inhibitors, leading to the dissociation of the inhibitor/NFkB complex and ultimately the degradation of the inhibitor [51]. Our finding shows that there is a disturbed expression of activators and repressors of the NFkB complex. Further studies are necessary to understand the actual role of NFkB in TLE(HS).

## 5. Conclusions

We have demonstrated the upregulation of various genes associated with the apoptosis through death receptor signaling in the hippocampus from TLE(HS) patients. The expression of the vast majority of these genes was studied for the first time in hippocampal human samples despite previous protein results, mainly from seizure models. Our data reveals that both TNF receptor genes (*TNFRSF1A* and* TNFRSF1B*) are overexpressed in patients. As key factors in the TNF-induced apoptosis, they may play a crucial role in hippocampal neuroinflammation and neurodegeneration.

Additionally, our results clearly suggest that three different death receptor apoptotic systems may be associated with the maintenance and progression of TLE-associated HS: (1) TNF-TNFRSF1A, (2) FASLG-FAS, and (3) TNFSF10-TNFRSF10A/B. Their effects on epilepsy are still scarcely comprehended. Other targets, such as the NFkB subunits and regulators or the decoy receptor of FAS, TNFRSF6B, whose gene upregulation has never been associated with epilepsy, should be investigated. The understanding of these signaling pathways is essential for the development of new antiepileptic drugs and opens several possible avenues of research that will help us to understand the complex pathophysiology in HS.

## Figures and Tables

**Table 1:** Array target genes: apoptosis through death receptors in TLE(HS).

Target genes			
Symbol, approved name, and Assay ID	Synonyms and aliases	Function	RQ
ACTA1 actin, alpha 1, skeletal muscle Hs00559403_m1	NEM3, “nemaline myopathy type 3”	Cell motility	1.12

ACTB actin, beta Hs99999903_m1		Cell motility	1.74

APAF1 apoptotic peptidase activating factor 1 Hs00559441_m1	APAF-1, CED4	Apoptosis induction by CASP3 activation	1.59

BAX BCL2-associated X protein Hs00180269_m1	BCL2L4	Apoptosis induction by CASP3 activation	1.50

BCL2 B-cell CLL/lymphoma 2 Hs99999018_m1	Bcl-2, PPP1R50, “protein phosphatase 1, and regulatory subunit 50”	Apoptosis inhibition	1.87

BID BH3 interacting domain death agonist Hs00609632_m1		Apoptosis induction	1.40

BIRC2 baculoviral IAP repeat containing 2 Hs01112284_m1	“Apoptosis inhibitor 1”, c-IAP1, cIAP1, hiap-2, MIHB, “NFR2-TRAF signalling complex protein”, and RNF48	Apoptosis inhibition	1.31

BIRC3 baculoviral IAP repeat containing 3 Hs00154109_m1	“Apoptosis inhibitor 2”, c-IAP2, cIAP2, hiap-1, “inhibitor of apoptosis protein 1”, MALT2, “mammalian IAP homolog C”, MIHC, RNF49, and “TNFR2-TRAF signaling complex protein”	Apoptosis inhibition	1.27

CASP2 caspase 2 Hs00234982_m1	ICH1, MGC2181, PPP1R57, and “protein phosphatase 1, regulatory subunit 57”	Involved in the activation cascade of caspases responsible for apoptosis execution	1.38

CASP3 caspase 3 Hs00234387_m1	Apopain, CPP32, CPP32B, and Yama	Involved in the activation cascade of caspases responsible for apoptosis execution	1.44

CASP7 caspase 7 Hs00169152_m1	CMH-1, ICE-LAP3, and MCH3	Involved in the activation cascade of caspases responsible for apoptosis execution; overexpression promotes programmed cell death	2.13

CASP8 caspase 8 Hs01018151_m1	Casp-8, FLICE, MACH, and MCH5	Most upstream protease of the activation cascade of caspases responsible for the FAS mediated and TNFRSF1A induced cell death	2.45

CASP9 caspase 9 Hs00154260_m1	APAF-3, ICE-LAP6, MCH6, PPP1R56, and “protein phosphatase 1, regulatory subunit 56”	Involved in the activation cascade of caspases responsible for apoptosis execution	1.55

CFLAR CASP8 and FADD like apoptosis regulator Hs01116280_m1	c-FLIP, CASH, Casper, CLARP, FLAME, FLIP, I-FLICE, and MRIT	Apoptosis regulator protein as FAS mediated apoptosis inhibitor	1.59

CHUK conserved helix-loop-helix ubiquitous kinase Hs00175141_m1	IKBKA, IKK-alpha, IKK1, IKKA, and NFKBIKA	Serine kinase. Essential role in the NFkB signaling pathway	1.22

DAXX death-domain associated protein Hs00154692_m1	DAP6	JNK pathway and apoptosis mediator via MAP3K5 [FAS and TGFBR2 (transforming growth factor beta receptor II) signaling]	1.53

DIABLO diablo, IAP-binding mitochondrial protein Hs00219876_m1	DFNA64, DIABLO-S, FLJ10537, FLJ25049, “second mitochondria-derived activator of caspase”, and SMAC	Apoptosis promoter by caspase activation in the cytochrome c/APAF1/CASP9 pathway	1.45

FADD Fas associated via death domain Hs00538709_m1	“Fas-associating death domain-containing protein,” “Fas-associating protein with death domain,” GIG3, “growth-inhibiting gene 3 protein,” “mediator of receptor-induced toxicity,” and MORT1	CASP8 and CASP10 apoptotic adaptor recruiter to activated FAS and TNFRSF1A	1.13

FAS Fas cell surface death receptor Hs00531110_m1	APO-1, CD95, and “TNF receptor superfamily member 6”	Receptor with death domain for FASLG	1.65

FASLG Fas ligand Hs00181225_m1	CD178, FasL	Cytokine ligand for FAS	5.46

IKBKB inhibitor of kappa light polypeptide gene enhancer in B-cells, kinase beta Hs00233287_m1	IKK-beta, IKK2, IKKB, and NFKBIKB	NFkB activator	1.96

IKBKG inhibitor of kappa light polypeptide gene enhancer in B-cells, kinase gamma Hs00415849_m1	FIP-3, FIP3, Fip3p, IKK-gamma, NEMO, and ZC2HC9	NFkB activator	1.65

MAP3K5 mitogen-activated protein kinase kinase kinase 5 Hs00178726_m1	“Apoptosis signal regulating kinase 1,” ASK1, and MAPKKK5	Signal transduction mediator by oxidative stress and receptor-mediated inflammatory signals (TNF)	1.55

MAPK8 mitogen-activated protein kinase 8 Hs01548508_m1	JNK, JNK1, “JUN N-terminal kinase,” and SAPK1	Stressed cell apoptosis promoter through TP53 and YAP1	1.05

MAPK9 mitogen-activated protein kinase 9 Hs00177102_m1	JNK2, “Jun kinase,” p54a, and SAPK	Stressed cell apoptosis promoter through TP53 and YAP1	0.86

NFKB1 nuclear factor of kappa light polypeptide gene enhancer in B-cells 1 Hs00765730_m1	KBF1, NF-kappaB, NF-kB1, NFkappaB, NFKB-p50, p105, and p50	Rel protein-specific transcription inhibitor (105 kD) and DNA binding subunit of the transcription factor NFkB (50 kD)	1.50

NFKB2 nuclear factor of kappa light polypeptide gene enhancer in B-cells 2 (p49/p100) Hs00174517_m1	LYT-10, NF-kB2, p105, and p52	Subunit of the transcription factor NFkB	3.21

NFKBIA nuclear factor of kappa light polypeptide gene enhancer in B-cells inhibitor, alpha Hs00153283_m1	IkappaBalpha, IKBA, and MAD-3	NFkB inhibitor	2.36

NGF nerve growth factor (beta polypeptide) Hs01113193_m1		Ligand for NTRK1 and NGFR; neuronal proliferation, differentiation and survival regulator	0.90

NGFR nerve growth factor receptor Hs00609976_m1	CD271, “low affinity nerve growth factor receptor,” p75NTR, “TNFR superfamily, member 16,” and TNFRSF16	Neural cell death or survival mediator	3.53

PARP1 poly(ADP-ribose) polymerase 1 Hs00242302_m1	PARP	DNA repair pathway initiation; apoptosis promoter in response to genotoxic stress	1.51

RELA v-rel avian reticuloendotheliosis viral oncogene homolog A Hs00153294_m1	p65	Subunit of the transcription factor NFkB	2.02

TNF tumor necrosis factor Hs00174128_m1	DIF, “TNF superfamily, member 2,” TNF-alpha, and TNFSF2	Multifunctional proinflammatory cytokine ligand for TNFRSF1A and TNFRSF1B	39.96

TNFRSF10A tumor necrosis factor receptor superfamily member 10a Hs00269492_m1	Apo2, CD261, DR4, and TRAILR-1	Receptor with death domain for TNFSF10; NFkB activator	2.39

TNFRSF10B tumor necrosis factor receptor superfamily member 10b Hs00366278_m1	CD262, DR5, KILLER, TRAIL-R2, TRICK2A, and TRICKB	Receptor with death domain for TNFSF10; ER stress-induced apoptosis promoter; NFkB activator	1.55

TNFRSF10C tumor necrosis factor receptor superfamily member 10c Hs00182570_m1	CD263, DcR1, LIT, TRAILR3, and TRID	Decoy receptor for TNFSF10; apoptosis inhibitor	4.82

TNFRSF10D tumor necrosis factor receptor superfamily member 10d Hs00533560_m1	CD264, DcR2, TRAILR4, and TRUNDD	Decoy receptor for TNFSF10; apoptosis inhibitor	3.74

TNFRSF1A tumor necrosis factor receptor superfamily member 1A Hs00205419_m1	CD120a, TNF-R, TNF-R-I, TNF-R55, TNFAR, and TNFR60	Receptor with death domain for TNF and LTA (lymphotoxin alpha); NFkB activator, apoptosis mediator, and inflammation regulator	3.07

TNFRSF21 tumor necrosis factor receptor superfamily member 21 Hs00237054_m1	CD358, “death receptor 6,” and DR6	Receptor with death domain; apoptosis promoter	1.09

TNFRSF25 tumor necrosis factor receptor superfamily member 25 Hs00187070_m1	APO-3, DDR3, DR3, LARD, TR3, TRAMP, WSL-1, and WSL-LR	Receptor with death domain for TNFSF12; mediator of NFkB activation and apoptosis promoter	1.72

TNFRSF6B tumor necrosis factor receptor superfamily member 6b Hs00234356_m1	DcR3, DCR3, M68, and TR6	Decoy receptor for FASLG; apoptosis inhibitor	2.54

TNFSF10 tumor necrosis factor superfamily member 10 Hs00174664_m1	Apo-2L, CD253, TL2, and TRAIL	Cytokine ligand for TNFRSF10A, TNFRSF10B, TNFRSF10C, and TNFRSF10D	2.50

TP53 tumor protein p53 Hs01034249_m1	LFS1, “Li-Fraumeni syndrome,” and p53	Tumor suppressor, growth arrest, or apoptosis promoter depending on the physiological circumstances and cell type	2.77

TRAF2 TNF receptor associated factor 2 Hs00184192_m1	TRAP3	Mediator of the antiapoptotic signals from TNF receptors; NFkB and JNK activation regulator	1.54

List of all target genes investigated in the array, involved in the extrinsic apoptotic pathway. The gene expression data [relative quantification (RQ) column], also shown in Figure 1, corresponds to the cDNA pooled from TLE(HS) patients (*n* = 12) versus the cDNA pooled from post mortem controls (*n* = 4). The latter was used as a calibrator with gene expression equal to 1.0. The values in the table can be converted to fold change values, where the negative inverse (−1/*x*) is taken for values between 0 and 1 (e.g., 0.5 is converted to −2). Values greater than 1 will not be affected. A −2 value indicates that the molecule is 2-fold downregulated. Only *MAPK9* and *NGF* were downregulated. The reference gene candidates in the plate were 18S (eukaryotic 18S rRNA; Hs99999901_s1), *GAPDH* (glyceraldehyde-3-phosphate dehydrogenase; Hs99999905_m1), *GUSB* (glucuronidase, beta; Hs99999909_m1), and *HPRT1* (hypoxanthine phosphoribosyltransferase 1; Hs99999908_m1) although only *GAPDH* and *HPRT1* were effectively used as reference genes (in combination).

## Abbreviations

BAX: BCL2-associated X protein
BID: BH3 interacting domain death agonist
CASP: Caspase
ENO2: Enolase 2 (gamma, neuronal)
FAS: Fas cell surface death receptor
HPRT1: Hypoxanthine phosphoribosyltransferase 1
HS: Hippocampal sclerosis
JNK: c-Jun N-terminal kinase
NIK: NFkB-inducing kinase
RT-qPCR: Reverse Transcription-Quantitative PCR
tBID: Truncated BID
TBP: TATA-box binding protein
TLE(HS): Temporal lobe epilepsy associated with hippocampal sclerosis
TLE: Temporal lobe epilepsy
TNF: Tumor necrosis factor
TNFRSF10A: Tumor necrosis factor receptor superfamily, member 10a
TNFRSF10B: Tumor necrosis factor receptor superfamily, member 10b
TNFRSF1A: Tumor necrosis factor receptor superfamily, member 1a
TNFRSF21: Tumor necrosis factor receptor superfamily, member 21
TNFRSF25: Tumor necrosis factor receptor superfamily, member 25
TP53: Tumor protein p53
TRAF2: TNF Receptor-Associated Factor 2
TRAFs: TNF Receptor-Associated Factors
TRAIL: TNF-related apoptosis-inducing ligand
XIAP: X-linked inhibitor of apoptosis.
